# Les lésions histopathologiques du placenta au cours de retard de croissance intra utérine sévère: à propos d'un cas clinique

**DOI:** 10.11604/pamj.2019.34.56.15160

**Published:** 2019-09-27

**Authors:** Hanaa Zaidi, Najat Lamalmi, Abderahmane Malihi, Amina Barkat, Zaytouna Alhamany

**Affiliations:** 1 Université Mohammed V, Faculté de Médecine et de Pharmacie, 10170, Rabat, Maroc; 2 Laboratoire d'Anatomie et de Cytologie Pathologique du Complexe Hôpital d'Enfant Maternité Souissi CHU Ibn Sina, 10170, Rabat, Maroc; 3 Service de Néonatologie P5, Hôpital d'Enfant CHU Ibn Sina, 10170, Rabat, Maroc

**Keywords:** Placenta, retard de croissance intra-utérin, examen placentaire, diagnostic histologique, prévention, Placenta, intra-uterine growth retardation, placenta examination, histological diagnosis, prevention

## Abstract

L'examen du placenta est indispensable dans le diagnostic du retard de croissance intra-utérin (RCIU). Son intérêt réside dans la recherche étiologique de cette pathologie, et les conséquences materno-fœtal qui peuvent en découler, ainsi dans la mise en place des stratégies préventive lors des grossesses ultérieures dans le cas des pathologies récidivantes. Nous rapportons les lésions anatomopathologiques possibles retrouvées au niveau du placenta de cette pathologie à travers une observation de RCIU sévère suivi au sein du Service de Néonatologie du CHU Ibn Sina de Rabat.

## Abstract (en)

The examination of the placenta is part of the assessment of intrauterine growth retardation (IUGR). Its interest lies in retardation etiology research and in maternal-fetal consequences which can arise from it as well as in the implementation of preventive strategies for subsequent pregnancies in patients with recurrent diseases. We conducted a study of patients with severe IUGR monitored in the Department of Neonatology of the Ibn Sina University Hospital in Rabat in order to identify possible anatomopathological lesions in the placenta.

## Introduction

Le retard de croissance intra-utérin (RCIU) est caractérisé par une cassure de la courbe de croissance fœtale, aboutissant habituellement à un poids de naissance trop faible pour l'âge gestationnel, généralement inférieur au 10^ème^ percentile (10% de poids de la naissance), et parfois il est sévère inférieur au 3^ème^ percentile (3% de naissance) [1, 2]. Elle représente une cause majeure de morbidité et de mortalité périnatales. L'examen anatomopathologique du placenta est indispensable dans les cas de RCIU. Son intérêt réside dans le diagnostic étiologique de cette pathologie afin de pouvoir mettre en place des mesures préventives lors des grossesses ultérieures. En effet cet examen détecte le plus souvent une origine vasculaire, ou parfois une origine infectieuse (villite chronique) [3]. Nous allons illustrer l'intérêt de l'examen anatomopathologique du placenta à travers une observation de RCIU sévère dont le placenta a été examiné dans notre laboratoire d'Anatomie et Cytologie Pathologique du CHU Ibn Sina de Rabat.

## Patient et observation

### Renseignements clinique

Madame J.B, âgée de 33 ans, G2P1, elle n'est pas connue porteuse d'une pathologie générale type diabète et/ou hypertension artérielle. Elle a eu comme antécédent obstétrical une première grossesse arrêtée à 45 jours. La 2^ème^ grossesse était bien suivie avec découverte à 29 SA d'une souffrance fœtale et RCIU sévère. À 35 SA la patiente a été admise pour fièvre à 39°C avec RCIU et anamnios. L'examen clinique a retrouvé une tension artérielle (TA) à 14/09, et le bilan biologique a montré un taux de globules rouges de 4,96/mm^3^, de globules blancs de 4700/mm^3^ et le taux des plaquettes de 86000/mm^3^. La C-réactive protéine (CRP) était de 24 mg/L, le taux de prothrombine (TP) à 100%, et la glycémie à jeûn était de 0,64g/l, Transaminase (ASAT) et Lactate Deshydrogénase (LDH) étaient respectivement de 102 UI/l et 240 UI/l. Devant le tableau clinique et l'aspect échographique (Anamnios+RCIU) une césarienne a été réalisée en urgence. En peropératoire la maman a présenté un pic hypertendu de 18/07 et des signes neurosensoriels. Avec à l'ouverture de l'utérus la découverte d'un hématome retro-placentaire (HRP) occupant 1/3 de la surface du placenta. La patiente a été admise en réanimation en post-partum immédiat. Au bloc opératoire un nouveau-né de sexe féminin d'aspect rose non déshydraté a été extrait à un poids nettement inférieur à la normale 950g (2100g). Apgar à la naissance était de 7/10 puis à 10/10 à la cinquième minute. La taille était de 34cm (46cm), périmètre crânien (PC) était de 26,5cm (32cm), la température était de 36°C. Ses mensurations correspondants à un âge gestationnelle de 27 à 28 SA. Le nouveau-né a été admis en réanimation néonatale pour prise en charge de prématurité et RCIU sévère. À J11 le poids du nouveau-né atteint 1020g, puis 1350g à J30 de son hospitalisation. L'enfant est suivi régulièrement dans une autre ville. À l'âge de 12 mois le poids atteint 8 Kg, avec un bon développement psychomoteur.

### Examen anatomopathologique du placenta

#### Examen macroscopique

**Figure 1:**
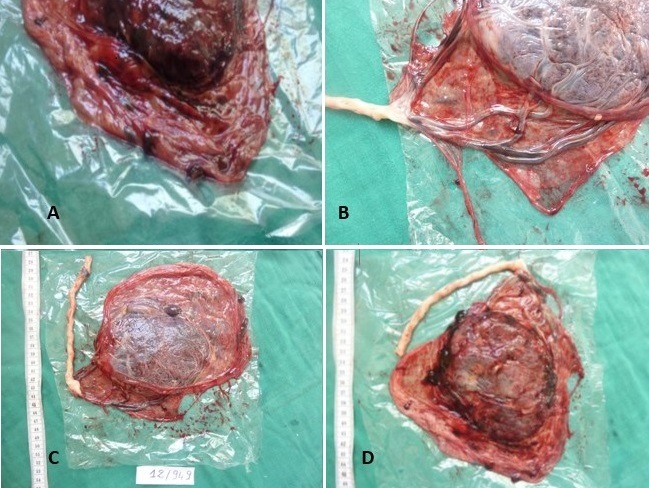
Aspect du placenta à l´état frais: (A) aspect congestif des membranes; (B) insertion vélamenteuse du cordon; (C) aspect modifiée de la plaque choriale avec des thromboses sous choriales, (D) aspect hétérogène de la plaque basale avec un hématome déciduale marginale

**Figure 2:**
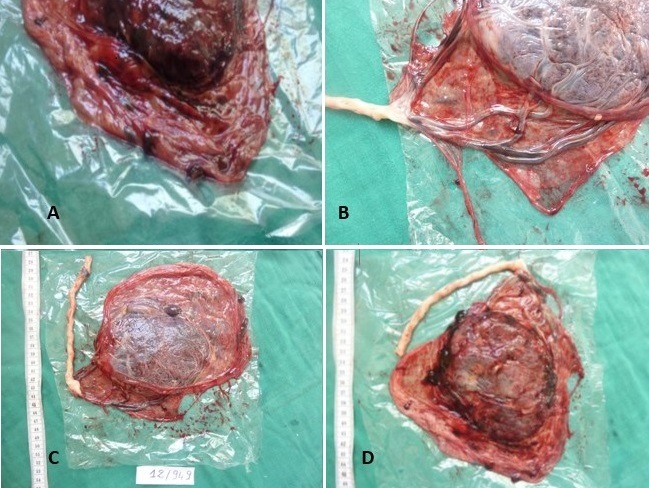
Placenta après fixation coupée en tranches sériées présentant des nombreux infarctus d´âge différent; parenchyme lésionnel estimé à > 30% du parenchyme placentaire

Après la délivrance le placenta a été envoyé au Laboratoire d'Anatomie et Cytologie Pathologique de l'Hôpital d'Enfants du CHU Ibn Sina de Rabat. Le placenta était de configuration ovalaire, pesait 220g (384g) et mesurait 13,5 sur 12cm (19cm) et 2,5 à 1,5cm d'épaisseur. Les membranes étaient insérées normalement, d'aspect congestif. Le cordon restant était d'insertion velamenteuse, grêle, mesurait 17cm de longueur et 1cm de diamètre, comportait 3 vaisseaux. La plaque choriale était d'aspect peu modifié avec des thromboses sous-choriale étendus. La plaque basale était d'aspect hétérogène avec un hématome déciduale marginale non adhérent sans dépression cupuliforme. En autre on a noté une zone épaisse et blanchâtre renfermant des calcifications (Figure 1). Après 24h de fixation au formol tamponné à 10%, l'examen des tranches de sections réalisées a retrouvé de nombreux foyers anciens et récents de taille variable de 1 à 2cm. Ainsi l'existence des lésions multiples étaient estimé à plus de 30% de parenchyme placentaire (Figure 2). Les lésions de nécrose ischémique avec dépôt de substance fibrinoide (NIDF) sont nombreuses. Plusieurs prélèvements sont effectués en tissu apparent macroscopiquement normale et en zones pathologique.

#### Examen histologique

**Figure 3:**
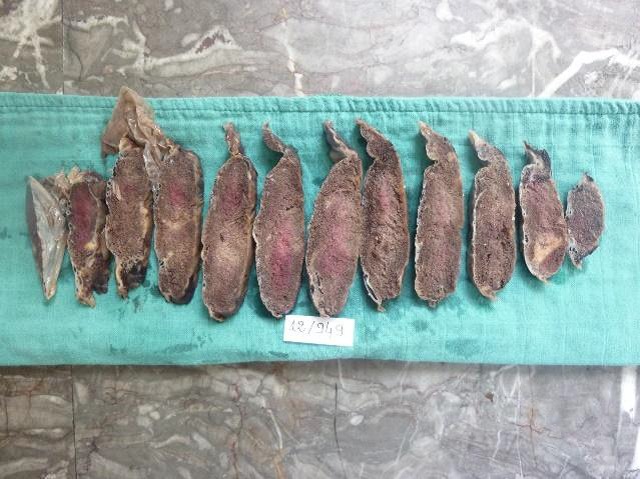
(A) lésions histologique du placenta; (B) les infarctus récents et anciens; (C) des thromboses de vaisseaux chorioallantoïdiens avec une partie de membrane à ce niveau; (D) une intense congestion des villosités avec une augmentation des sections vasculaires au niveau de la zone du cotylédon

Les lésions constatées macroscopiquement blanchâtres étaient des zones des infarctus d'âge différents avec un hématome déciduale marginale. Les infarctus anciens sont représentés par des villosités tassées les unes contre les autres avasculaires et cernées par une couronne de substance fibrinoide qui occupe l'espace intervilleux (Figure 3A, Figure 3B). Dans le parenchyme normal les villosités sont en nombre et en taille petites par rapport à l'âge gestationnelle. Il n'y a pas de foyer de villite chronique. On note aussi des amas nucléaires syncithiaux en excès. Au niveau de la plaque choriale il existe des thromboses de vaisseaux chorioallantoïdiens (Figure 3C) Sur la zone de cotylédon apparent normale on note une intense congestion des villosités avec une augmentation des sections vasculaires: chorioangiose (Figure 3D) Les dépôts de NIDF sont trop abondants pour le terme. Au terme de l'examen anatomopatologique du placenta on retient: placenta de petit poids (220g) d'insertion vélamenteuse du cordon signant une hypotrophie placentaire sévère avec la présence d'un hématome décidual marginal associé à de nombreux infarctus (> 30% de parenchyme lésionnel), et des lésions de NIDF, correspondant à un défaut de perfusion placentaire responsable du retard staturo-pondéral sévère de l'enfant. Il n'a pas été vu d'autre anomalie associée.

## Discussion

Le retard de croissance intra-utérin (RCIU) est une cause fréquente de consultation anténatale. Le pronostic dépend de l'étiologie: vasculaire, chromosomique, génétique, ou infectieuse. L'hypotrophie augmente les risques de mortalité, de morbidité et de séquelles neurosensorielles [4]. Par ailleurs, certaines causes du RCIU sont responsables de récidives lors des grossesses ultérieures. L'idéal est donc de connaître l'étiologie du RCIU afin de pouvoir mettre en place des mesures préventives lors des grossesses ultérieures. L'examen du placenta fait partie du bilan du RCIU. Son intérêt réside dans la recherche étiologique et les conséquences potentielles pédiatriques ou maternelles qui peuvent en découler. L'étiologie la plus fréquemment retenue devant un RCIU est l'insuffisance vasculaire placentaire d'origine maternelle. Ce diagnostic n'est cependant pas simple à l'examen anatomopathologique du placenta car il n'y pas de lésion pathognomonique mais un faisceau d'arguments diagnostiques. Cependant, les lésions morphologiques compatibles avec l'insuffisance vasculaire d'origine maternelle sont les plus fréquemment mises en évidence sur l'ensemble des placentas examinés en anatomie pathologique toutes indications confondues. Des études récentes montrent que l'atteinte vasculaire maternelle est classiquement considérée comme la cause la plus fréquente de RCIU [5-7]. Ainsi des études faites par Redline et Aviram *et al.* ont porté chacune sur une centaine de cas et ont conclu à une cause vasculaire maternelle dans respectivement 61 et 66% des placentas de RCIU [8, 9]. Les lésions histopathologiques placentaire retrouvé dans notre cas sont compatible avec les études de Redline, définit et décrit l'insuffisance d'apport vasculaire maternel par une mauvaise implantation et ses conséquences: des anomalies de configuration placentaire, des anomalies de l'insertion du cordon; une obstruction partielle des vaisseaux maternels d'où des amas syncytio nucléaires en excès, des villosités tassées entourées de fibrine ou bien une obstruction complète d'un vaisseau et sa conséquence: l'infarctus; Une rupture de paroi d'un vaisseau maternel soit de type artériel: hématome décidual basal, soit de type veineux: hématome décidual marginal [10].

Dans l'examen macroscopique du placenta reporté dans notre observation, il y avait de nombreuses lésions associé au RCIU sévère d'origine vasculaire maternelle L'hypotrophie placentaire présentée dans notre observation par un poids de 220g, avec prédominance villositaire, des villosités petites, clairsemées et des espaces intervillositaires larges, est fréquemment associé à un RCIU [11, 12]; Dans des autres études l'association hypotrophie placentaire et RCIU constitue le cas habituel. Le placenta peut être de petit poids sans lésion vasculaire ou plus fréquemment de petit poids avec des lésions macroscopiques et/ou microscopiques comme chez notre cas [5-7]. Dans une cohorte de 6410 placentas de Kajantie *et al.* La configuration ovale du placenta est plus fréquente en cas de pathologie vasculaire maternelle et de prééclampsie [13]. Dans l'étude rétrospective de Rainassen *et al.* réalisée à partir de 26000 naissances consécutives, l'insertion vélamenteuse du cordon survient dans 1 à 2% des cas. Elle serait statistiquement corrélée à l'insuffisance vasculaire placentaire d'origine maternelle (p < 0,003), aux enfants avec RCIU (p < 0,001) par comparaison aux témoins avec cordon d'insertion normale [14]. Au niveau histologique les infarctus d'âges déférents sont fréquemment associés à la prééclampsie sans être un signe pathognomonique. Le pourcentage de parenchyme atteint donne une idée de la dysfonction placentaire [7]. Dans notre observation le parenchyme lésionnel a été estimé à plus de 30%. L'étude rétrospective cas-témoins de Vinnars *et al.* Sur 157 cas de prééclampsie a montré des infarctus d'autant plus nombreux que la prééclampsie est sévère: 39,7% des placentas ont plus de 5% de parenchyme infarci en cas de prééclampsie sévère, 17,1% en cas de prééclampsie modérée et 5,1% sans prééclampsie [15]. Dans une autre étude cas-témoins, la présence d'infarctus à terme est trouvée corrélée à l'hypertension maternelle que ce soit chez les enfants de poids normal (OR = 2,99; IC95 %: 1,23-7,32) ou chez les enfants porteur d'un RCIU (OR = 4; IC95 %: 1,96-8,16) [16]. Aussi au niveau histologique il y avait de nombreuses lésions de NIDF, ce type de lésion est associé à un RCIU. Dans la série de Katzman, 31% (11/36) des cas de NIDF centrale sont associées à un RCIU [17] Ces lésions intéressent souvent plus de 25% des villosités et sont associées à un RCIU souvent précoce, Cette lésion est considérée comme étant une anomalie de la tolérance materno-foetale avec un risque de récurrence élevé [18]. Dans le parenchyme placentaire, la réponse adaptative à l'hypoperfusion chronique de début précoce se manifeste par plusieurs lésions élémentaires microscopiques dont aucune n'est spécifique. Ces lésions sont de développement villositaire: absence de villosités inter-médiaires et hypoplasie des villosités terminales, la maturation avancée des villosités, les amas syncytiaux en excès, le défaut tassement des villosités les unes contre les autres, la présence de fibrine en excès, l'érythroblastose [6, 7, 19, 20]. Les amas syncytiaux en excès forment la lésion microscopique la plus fréquente dans le cas de prééclampsie [6, 7]. L'étude cas-témoins de Devisme *et al.* a montré la présence d'amas syncytiaux dans 90% des placentas de prééclampsie et dans seulement 9,2% des témoins [21]. La vasculopathie fœtale thrombotique, décrites par la présence des villosités avasculaires sont associées au RCIU sévère, [22]. Ces lésions s'observent exclusivement à la fin du 2^ème^ trimestre et au 3^ème^ trimestre. La chorangiose villositaire est une lésion histologique présenté dans notre observation intéresse uniquement les villosités terminales et est définie par la présence de plus de 10 capillaires dans les villosités terminales adjacentes [23]. Cette lésion adaptative est supposée être corrélée à l'hypoxie chronique et au RCIU mais ne serait pas associée à la prééclampsie [5, 24]. Le risque de récidive en cas de RCIU avec prééclampsie modérée est de 10%, et de 30 à 40% en cas de RCIU précoce et sévère d'origine vasculaire [25].

## Conclusion

L'examen anatomopathologique du placenta est indispensable en cas de retard de croissance intra-utérin et la souffrance fœtale, il permet à la fois d'établir un diagnostic étiologique pour une prise en charge adéquat du nouveau-né, et la prévention des récidives pour les grossesses ultérieures.

## Conflits d’intérêts

Les auteurs ne déclarent aucun conflit d'intérêts.

## References

[1] Gabriel R, Harika G (2008). Retard de croissance intra-utérin et fœtus petits pour l'âge gestationnel. Revue francophone des laboratoires.

[2] French College of Gynecologists and Obstetricians (2013). Intra-uterine growth retardation: guidelines for clinical practice-short text. J Gynecol Obstet Biol Reprod (Paris).

[3] Marcorelles P (2013). Placental features in intrauterine growth retardation. J Gynecol Obstet Biol Reprod (Paris).

[4] Cordier A-G, Nedellec S, Benachi A, Frydman R, Picone O (2011). Arguments for an infectious cause of IUGR. J Gynecol Obstet Biol Reprod (Paris).

[5] Cox P, Marton T (2009). Pathological assessment of intrauterine growth restriction. Best Pract Res Clin Obstet Gynaecol.

[6] Benirschke K, Kaufman P (2000). Pathology of the human placenta.

[7] Kraus F, Redline RW, Gersell DJ, Nelson DM, Dicke JM (2004). Placental Pathology. Atlas of non tumoral pathology.

[8] Redline RW, O'Riordan MA (2000). Placental lesions associated with cerebral palsy and neurologic impairment following term birth. Arch Pathol Lab Med.

[9] Aviram R, Shental B, Kidron D (2010). Placental aetiologies of foetal growth restriction: clinical and pathological differences. Early Hum Dev.

[10] Redline RW (2008). Placental pathology: a systematical approach with clinical correlations. Placenta.

[11] Fournie A, Kessier S, Biquard F, Parant O, Connan L (2004). Hypotrophie, retard de croissance intra-utérin, souffrance fœtale chronique. EMC (Elsevier SAS, Paris) obstétrique.

[12] Hermine-Coulomb AL (2005). Examen du placenta. EMC (Elsevier SAS, Paris) obstétrique.

[13] Kajantie E, Thornburg KL, Erikson JG, Osmond C, Barker DJP (2010). In preeclampsie, the placenta grows slowly along its minor axis. Int J Dev Biol.

[14] Raisanen S, Georgiadis L, Harju M, Keski-Nisula L, Heinonen S (2012). Risk factors and adverse pregnancy outcomes among births affected by velamentous umbilical cord insertion: a retrospective population-based register study. Eur J Obstet Gynecol Reprod Biol.

[15] Vinnars MT, Nasiell J, Ghazi S, Westgren M, Papadogiannakis N (2011). The severity of clinical manifestations in preeclampsia correlates with the amount of placental infarction. Acta Obstet Gynecol Scand.

[16] Bencroft D, Thompson JM, Mitchell EA (2004). Placental infarcts, intervillous fibrin plaques and intervillous thrombi: incidences, cooccurences and epidemiological associations. Pediatr Dev Pathol.

[17] Bada HS, Das A, Bauer CR, Shankaran S, Lester BM, Gard CC, Wright LL, Lagasse L, Higgins R (2005). Low birth weight and preterm births: etiologic fraction attributable to prenatal drug exposure. J Perinatol.

[18] Katzman PJ, Genest DR (2002). Maternal floor infarction and massive perivillous fibrin deposition: histological definitions, associations with intrauterine fetal growth restriction, and risk of recurrence. Pediatr Dev Pathol.

[19] Redline RW, Heller D, Keating S, Kingdom J (2005). Placental diagnosis criteria and clinical correlation-a workshop report. Placenta.

[20] Ghidini A, Salafia CM, Pezzullo JC (1997). Placental vascular lesions and likelihood of diagnosis of preeclampsia. Obstet Gynecol.

[21] Devisme L, Merlot B, Ego A, Houfflin-Debarge V, Deruelle P, Subtil D (2013). A case-control study of placental lesions associated with preeclampsia. Int J Gynaecol Obstet.

[22] Redline RW, Pappin A (1995). Fetal thrombotic vasculopathy: the clinical significance of extensive avascular villi. Hum Pathol.

[23] Ogino S, Redline RW (2000). Villous capillary lesions of the placenta: distinction between chorangiomes, chorangiomatosis, and chorangiose. Hum Pathol.

[24] Roberts DJ, Post MD (2008). The placenta in pre-eclampsia and intra-uterine growth restriction. J Clin Pathol.

[25] Parant O (2002). Conduite à tenir devant une hypotrophie sévère. Jour-nées Pyrénéennes de gynécologie Tarbes.

